# Treatment with BRICHOS domain helps to clarify issues with Alzheimer mouse models

**DOI:** 10.1038/s44321-024-00041-1

**Published:** 2024-03-13

**Authors:** Jan Johansson

**Affiliations:** Department of Biosciences and Nutrition, Karolinska Institutet, Huddinge, Sweden

**Keywords:** Neuroscience

## Abstract

This correspondence offers clarifications on the utility of BRICHOS domains in understanding pathology in Alzheimer’s disease mouse models and the potential of these domains as a treatment strategy.

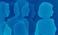

Mouse models of Alzheimer disease (AD) pathologies are hampered by potential confounding factors, such as site of transgene insertion, combination of mutations that have not been observed in patients, and overproduction of amyloid precursor protein and processing products. Results from recent treatments with the BRICHOS domain, with well-defined in vitro effects on amyloid-β peptide (Aβ) aggregation and neurotoxicity, help to identify likely molecular mechanisms in some AD mouse models.

Recently, D’Adamio reviewed strengths and weaknesses with currently available rodent models of different aspects of the pathologies associated with Alzheimer disease (AD), and discussed their relevance for advancing understanding of AD and related dementias (D’Adamio, 2023). Some of the mouse models discussed harbor the BRICHOS containing Bri2 protein. The BRICHOS domain is part of 10 human proprotein families that all contain a region with pronounced tendency to form β-sheet aggregates and amyloid fibrils, a potentially devastating scenario that the BRICHOS domain normally prevents (Leppert et al, 2023). The name BRICHOS derives from the three proteins first found to contain such a domain—*Bri*2, *Cho*ndromodulin and pro*s*urfactant protein C (proSP-C). The BRICHOS containing proprotein families share a common architecture and their BRICHOS domains have similar structures, but they are otherwise evolutionarily distant (Leppert et al, 2023). SP-C is one of the most amyloid-prone proteins known, and mutations in proSP-C BRICHOS domain lead to amyloid formation of the SP-C part and lethal disease in early childhood (Willander et al, 2012), whereas all other known amyloid diseases occur late in life. Thus these observations strongly suggest that the BRICHOS domain is a naturally occurring defense against toxic amyloid formation. Isolated, i.e., in the absence of the rest of the proproteins, BRICHOS domains from proSP-C and Bri2 can prevent amyloid fibril formation and the therewith associated cellular toxicity not only of its physiological clients but also of other amyloidogenic proteins, including AD relevant amyloid-β peptide (Aβ), see (Leppert et al, 2023) and islet amyloid polypeptide (IAPP) associated with type 2 diabetes (Oskarsson et al, 2018).

Published data using isolated recombinant human (rh) Bri2 BRICHOS domain for treatment of AD mouse models (Abelein and Johansson, 2023; Manchanda et al, 2023) may help to clarify some issues raised by D’Adamio (2023). One issue concerns the importance of Aβ versus other processing products of the Aβ precursor protein (APP) for development of AD like pathology. D’Adamio (2023) argues that the fact that mice which overproduce Aβ40 and Aβ42 from a Bri2–Aβ hybrid transgene, rather than from APP, do not show any cognitive impairment (Kim et al, 2013) suggests that other APP processing intermediates are required for effects on cognition. However, treatment of APP^NLF^ and APP^NLGF^ knock-in mice, which overproduce Aβ40 and Aβ42 but no other APP processing intermediates, develop AD relevant amyloid plaques, memory impairment, and neuroinflammation, but lacks tau pathology and neurodegeneration associated with AD (Saito et al, 2014), with intravenous rh Bri2 BRICHOS injections improves memory function along with reduced plaque load (Manchanda et al, 2023). Moreover, the fact that Bri2–Aβ hybrid transgenic mice also overproduce Bri2 BRICHOS offers another explanation than absence of non-Aβ processing intermediates for the lack of cognitive effects in these mice. In light of the literature data on Bri2 BRICHOS effects on Aβ aggregation and toxicity in vitro and well as in vivo, see above, it is conceivable that it actually is the production of Bri2 BRICHOS along with Aβ in the Bri2–Aβ hybrid transgenic mice that prevents cognitive decline. Further studies, for example by generating Bri2–Aβ hybrid transgenic mice that produce designed inactive Bri2 BRICHOS variants, see Chen et al (2022), along with Aβ may give important information both on the neurotoxic effects of Aβ and the protective effects of Bri2 BRICHOS.

Another issue discussed by D’Adamio (2023) is to what extent Aβ aggregation and toxicity actually underlie the pathology seen in AD models, as general confounding effects caused by, e.g., site of transgene insertion, accumulation of several mutations in APP and presenilins, and non-Aβ processing intermediates from APP are possible. Moreover, AD mouse models are limited as no mouse model that reproducibly replicates all the features of human AD pathology is known, while they show certain AD-associated pathologies, but lack others, see, e.g., D’Adamio (2023) and the comments on APP knock-in models above. Scrutiny of the treatment effects of intravenously injected rh Bri2 BRICHOS in APP^NLF^ and APP^NLGF^ knock-in mice shed some light also on the importance of Aβ aggregation and toxicity for development of some aspects of AD related pathology. Detailed analyses of the kinetics of Aβ42 fibril formation allows the estimation of the number of Aβ42 oligomers generated during the fibrillation process, which correlates well with observed toxic effects on hippocampal neural network activity in vitro, see, e.g., (Chen et al, 2020). Estimated reduction of Aβ42 oligomer generation and neurotoxicity that could be achieved by the rh Bri2 BRICHOS treatment scheme used (Manchanda et al, 2023) correlated very well with the treatment effects sizes on cognition, plaque load as well as neuroinflammation seen in the APP knock-in AD model mice (Abelein and Johansson, 2023). This strongly supports that similar mechanisms as have been shown for BRICHOS in vitro are relevant also in BRICHOS treated mice, which in turn implies that Aβ42 fibril formation and therewith associated neurotoxicity cause a significant part of the AD mouse model pathology.

The observations described herein suggest that molecular chaperones that have quantifiable effects on amyloid fibril formation and neurotoxicity, such as BRICHOS, are not only potentially useful for future treatment strategies but can also be harnessed to better understand molecular mechanisms of neurodegeneration.
